# Multilocus Analysis of Genetic Susceptibility to Myocardial Infarction in Russians: Replication Study

**Published:** 2017

**Authors:** N. G. Kukava, B. V. Titov, G. J. Osmak, N. A. Matveeva, O. G. Kulakova, A. V. Favorov, R. M. Shakhnovich, M. Ya. Ruda, O. O. Favorova

**Affiliations:** National Medical Scientific Center for Cardiology, 3rd Cherepkovskaya Str. 15a, Moscow, 121552 , Russia; Pirogov Russian National Research Medical University, Ostrovitjanova Str. 1, Moscow, 117997, Russia; Oncology Biostatistics and Bioinformatics, Johns Hopkins School of Medicine, Baltimore, MD 21205 , US

**Keywords:** myocardial infarction, Russians, genes, allelic polymorphism, multilocus analysis, genetic markers

## Abstract

In search of genetic markers of myocardial infarction (MI) risk, which have prognostic significance for Russians, we performed a replication study of MI association with genetic variants of *PCSK9 *(rs562556), *APOE *(epsilon polymorphism, rs7412 and rs429358), *LPL *(rs320), *MTHFR *(rs1801133), *eNOS *(rs2070744), and the 9p21 region (rs1333049) in 405 patients with MI and 198 controls. Significant MI association was observed with variants of the lipid metabolism genes (*PCSK9*, *APOE *and *LPL*), and of *eNOS*. The SNPs in the *MTHFR *gene and the 9p21 region were not significantly associated with MI one by one but were included in several different MI-associated allelic combinations identified by multilocus analysis. Since we have not revealed nonlinear epistatic interactions between the components of the identified combinations, we postulate that the cumulative effect of genes that form a combination arises from the summation of their small independent contributions. The prognostic significance of the additive composite model built from the *PCSK9*, *APOE*, *LPL, *and *eNOS *genes as genetic markers was assessed using ROC analysis. After we included these markers in the previously published composite model of individual genetic risk of MI, the prognostic efficacy in our sample reached AUC = 0.676. However, the results obtained in this study certainly need to be replicated in an independent sample of Russians.

## INTRODUCTION

Myocardial infarction (MI) is the most severe form of coronary artery disease (CAD). Although significant progress in prevention and treatment of cardiovascular diseases (CVDs) has been achieved over the past decades in the developed countries, MI still remains the leading cause of death worldwide.

Both MI and CAD are polygenic and multifactorial diseases; the non-Mendelian inheritance pattern characterizing them results from the interplay between genetic variants. The genetic predisposition to CAD has been well investigated in genome-wide association studies (GWAS), while the number of GWASs for MI as a particular phenotype is relatively small [1, 2]. The rather poor replicability of the few MI-associated loci identified in separate studies may be due to ethnic differences between the samples. Although the objective of the GWASs is to identify genetic variants that would enable assessing the risk of MI, no progress in predicting the risk of this disease has been made yet [3].

It is no wonder that the conventional candidate gene approach still remains relevant. Vast amounts of data have been accumulated on the association of individual candidate genes with MI in Russian population; much of these data have been obtained by Russian participants of the MONICA [4] and HAPIEE [5] international research projects. Replication of the results obtained both for the independent samples consisting of subjects belonging to the same ethnic group and other ethnic populations is believed to play a special role in identifying factors of genetic predisposition. We have earlier found that the genetic variants of the *FGB*, *TGFB1*, *CRP*, *IFNG*, and *PTGS1 *genes, whose products are involved in the inflammation and coagulation systems, are associated with the risk of MI development in ethnic Russians and replicated these results in an independent sample of Russians [6]. The prognostic significance of the identified markers has been demonstrated; summing up the contributions of individual genes significantly increased the prognostic efficacy. However, the identified loci explain only a small contribution to the risk of MI. In pursuit of other genetic markers for MI risk that would have prognostic significance for Russians, we have broadened the candidate gene list under study by including the lipid metabolism genes (*PCSK9*, *LPL*, and *APOE*), the *MTHFR *and the *eNOS *genes, and the 9p21 locus.

The products of the selected lipid metabolism genes are known to be involved in the development of CVDs. The PCSK9 protein (proprotein convertase subtilisin/ kexin type 9) encoded by the *PCSK9 *gene partakes in degradation of low-density lipoprotein receptors and is used as a target in treatment of dyslipidemia and related CVDs [7]. The *APOE *gene product, apolipoprotein E, is involved in lipid transport and plays a crucial role in the development of CVD [8]. Lipoprotein lipase encoded by the *LPL *gene is a key enzyme in lipid metabolism and transport; it also participates in pathogenesis of atherosclerosis [9].

The role of products of the *MTHFR *and *eNOS *genes in CVD pathogenesis is also well-known. The *MTHFR *gene codes for methylenetetrahydrofolate reductase, the enzyme involved in conversion of homocysteine to methionine. Homocysteinaemia may cause endothelial dysfunction, which is a risk factor for atherosclerosis and CVDs related to it [10]. Endothelial nitric oxide (NO) synthase encoded by the *eNOS *gene catalyzes production of NO involved in regulation of vascular tone and permeability; disturbances in the NO system may lead to atherosclerosis, hypertension, and thrombosis [11].

The MI association with the rs1333049 polymorphism on chromosome 9p21 was revealed in several GWASs and has been validated in a number of ethnic groups. This locus carries the gene of non-coding regulatory RNA ANRIL. This RNA may regulate the expression of cyclin-depended kinase inhibitors p15INK4a and p16INK4b, which are encoded by the *CDKN2A *and *CDKN2B *genes residing within the same region. It is believed that the 9p21 region can participate in pathogenesis of atherosclerosis by regulating proliferation and apoptosis of smooth muscle cells [12].

The aim of our study was to conduct a replication study of the association of the polymorphic variants in the *PCSK9*, *APOE, LPL*, *MTHFR*, and *eNOS *genes and the 9p21 region with the risk of MI development in Russians. *Table 1* lists the characteristics of the selected genes and single nucleotide polymorphisms (SNPs). We also carried out a multilocus analysis of the association between the combinations of variants of these genes/ loci and MI, since the cumulative genetic effect can be identified using this approach [13]. The nature of this effect was also studied. Furthermore, we evaluated the prognostic efficacy of the identified markers both one by one and along with the markers identified previously [6].

**Table 1:** Genes included in the study and their polymorphic regions

Gene	Chromosomal locus	Gene product(s)	Polymorphic region*	
			SNP	rs ID
PCSK9	1p32.3	Proprotein convertase subtilisin/kexin type 9	1420G > A	rs562556
APOE	19q13.2	Apolipoprotein E	epsilon polymorphism	(rs7412, rs429358)
LPL	8p22	Lipoprotein lipase	495T > G (HindIII H+ > H–)	rs320
MTHFR	1p36.22	5,10-Methylene tetrahydrofolate reductase	677C > T	rs1801133
eNOS (also known as NOS3)	7q36	Endothelial nitric oxide synthase	−786T > C	rs2070744
the ANRIL– CDKN2A/2B gene cluster	9p21.3	Long non-coding RNA (the ANRIL gene); cyclin-depended kinase inhibitors 2A and 2B (the CDKN2A and CDKN2V genes)	C > G	rs1333049

^*^The examined single nucleotide polymorphism (SNP) and its designation according to the reference nucleotide sequence of the human genome (rs ID).

## EXPERIMENTAL

Genomic DNA samples collected from patients receiving treatment at the Emergency Cardiology Department (National Medical Research Center of Cardiology, the Ministry of Health of the Russian Federation) were used in the case-control study. The study group consisted of 405 ethnic Russians (mean age (m. a.) ± standard deviation, 57.5 ± 12.8 years): 271 males (m.a., 53.4 ± 11.9 years) and 134 females (m.a., 65.6 ± 10.3 years). The diagnosis of MI was made using the criteria described in [14]. The control group consisted of 198 Russian subjects with no past history of CVD (mean age, 59.8 ± 13.3 years): 112 males (m.a., 57.1 ± 11.9 years) and 86 females (m.a., 63.2 ± 14.2 years). All patients provided informed consent for participating in the study.

**Genomic typing **was performed using the polymerase chain reaction (PCR)-based methods. Restriction fragment length polymorphism analysis of the PCR products was carried out to detect the *APOE *gene epsilon polymorphism (rs7412, rs429358) [15], 495T > G in an *LPL *gene (rs320) [16], 677C > T in the *MTHFR* gene (rs1801133) [17], and −786T>C in the *eNOS *gene (rs2070744) [18]. Genome typing of the polymorphisms rs562556 in the *PCSK9* gene and rs1333049 in the 9p21.3 region was performed by real-time PCR using a TaqMan® SNP Genotyping Assay kit (Applied Biosystems).

**Statistical analysis**

The deviations of genotype frequencies from the Hardy–Weinberg equilibrium were analyzed using Haploview 4.2 software [19]. APSampler software was used to search for the associations between carriage of alleles and genotypes of individual polymorphisms or their combinations and development of MI [20]. The significance of the revealed associations was assessed using the Fisher’s exact test and the odds ratio (OR). The Bonferroni correction for the number of tests (multiple comparisons) was used for the *p *values calculated using the Fisher’s exact test (*p_corr_*). The *p *and *p_corr_*values < 0.05 were considered significant when the 95% confidence interval (CI) values for OR did not cross 1. An SNP was considered to be myocardial infarction-associated when the association was significant either in the recessive or the dominant model.

The earlier proposed approach [6] was used to reveal possible non-linear interactions (epistasis) between alleles in the identified biallelic combinations: the synergy factor (SF) was determined [22] and the *p *values were calculated using the exact three-way interaction test [21]. The interaction between the alleles was considered to be epistatic if the *p *value was less than 0.05 and the 95% CI value for SF did not cross 1.

The prognostic models were built using the stepwise logistic regression method (Stats v.3.3.1 for R). The prognostic efficacy was assessed by ROC (receiver operating characteristic) analysis by measuring the area under the curve (AUC) using pROC v.1.8 for R software package; pairwise comparisons were made using the method described in [23]. The probability threshold was calculated using the procedure described in [24] to assess sensitivity and specificity of the prognostic models.

## RESULTS

**Fig. 1:**
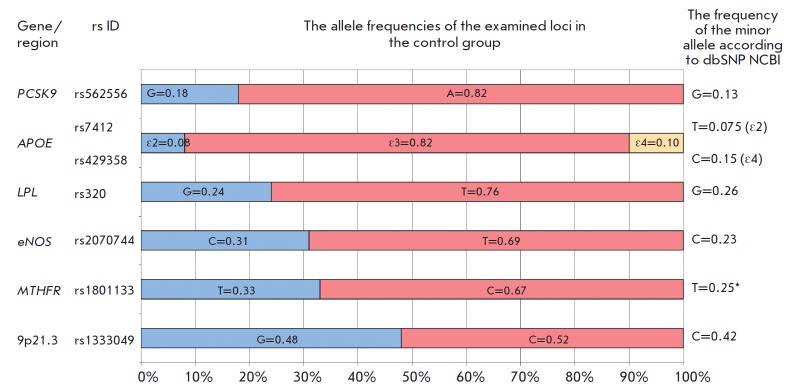
The allele frequencies of the examined loci in the control group (ethnic Russians) as compared to the allele frequencies from the dbSNP NCBI database [25]. *In the dbSNP NCBI database, minor allele frequency (MAF) is shown for the complementary chain.

All studied polymorphic regions in the control group were in Hardy–Weinberg equilibrium (*p* > 0.05). *Figure 1* shows the allele frequencies for all the examined loci in the control group compared to minor allele frequencies (global MAF) in the SNP Database under the 1000 Genomes Project (Phase 3) [25]. The absolute differences between the observed allele frequencies and those deposited in the SNP database are ≤ 10%.

*Table 2*summarizes the data on carriage of the alleles and genotypes of the *PCSK9 *(rs562556), *APOE *(epsilon polymorphism, rs7412 and rs429358), *LPL *(rs320), *MTHFR *(rs1801133), and *eNOS *(rs2070744) genes and the 9p21 region (rs1333049) in 405 MI patients and 198 controls*. *Significant differences were revealed in carriage frequencies of alleles and genotypes of polymorphic regions for all three lipid metabolism genes: *PCSK9*, *APOE, *and *LPL*. Significant differences were also found for the *eNOS *gene but not for the *MTHFR *gene or the 9p21 region. The PCSK9*A/A (*p *= 0.013, OR = 1.45), APOE*ε3/ε3 (*p *= 0034, OR = 1.52), and LPL* G/G (*p *= 0.032, OR = 1.96) genotypes and carriage of the eNOS*C allele (*p *= 0.0034, OR = 1.63) were found to be the risk factors of MI. However, the *pcorr *value calculated using the Bonferroni correction for the number of tests (multiple comparisons) was significant only for the genetic variants of the *APOE *and *eNOS *genes.

**Fig. 2:**
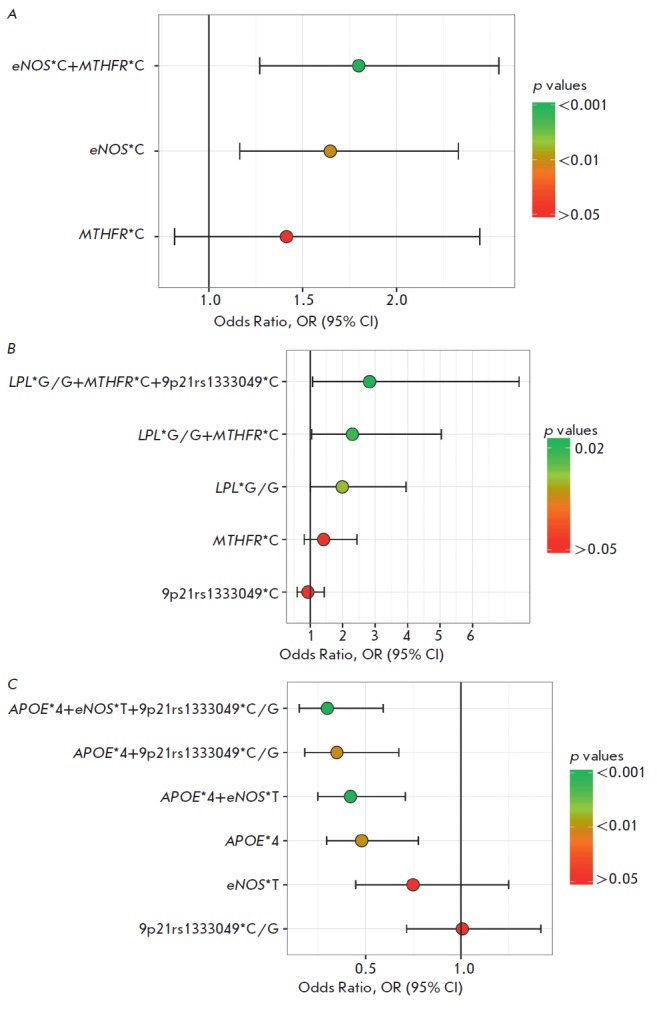
Multilocus analysis made it possible to identify the association between MI and rs1801133 in the *MTHFR *gene and rs1333049 in the 9p21 region, which one by one are not significantly associated with MI. The odds ratios (ORs), the confidence intervals (CIs) and the significance levels (qualitatively, by color of each circle, which corresponds to the OR value) are graphically presented for MI-associated combinations, which include the variants of the *MTHFR *gene and/or rs1333049, and for the components of these combinations. A. Biallelic combination (MTHFR*C + eNOS*C) that is positively associated with MI, and its components. B. Triallelic combination (LPL*G/G + MTHFR*C + rs1333049*C) that is positively associated with MI, and its components. C. Triallelic combination (APOE*ε4 + eNOS*T + rs1333049*C/G) that is negatively associated with MI, and its components.

We used the APSampler software employing the dynamic Monte Carlo method to carry out a multilocus analysis aimed at identifying the cumulative contribution of combinations of the alleles and genotypes of the genes under study to predisposition to MI. The revealed bi- and triallelic combinations associated with the risk of MI are characterized by a stronger effect and a greater significance level of association with MI than their individual components. Along with the *PCSK9*, *APOE, LPL, *and *eNOS *genetic variants, the combinations also include the alleles/genotypes of the *MTHFR *gene and SNP rs1333049 in the 9p21 region. *Figure 2* (*A–C*) illustrates the OR and 95% CI values for the combinations containing the variants of the latter two loci: MTHFR*C, rs1333049*C, and rs1333049*C/G. One can see in all these cases that the variants shown at the bottom of each figure are not significant. However, the combination of the MTHFR*C and eNOS*C alleles is significant (*p *= 0.0006; OR = 1.80): more significant than carriage of a single eNOS*C allele (*Fig. 2A*). The triallelic combination (LPL*G/G + MTHFR*C + rs1333049*C) (*p *= 0.018; OR = 2.83) and the biallelic combination being a part of it (LPL*G/G + MTHFR*C) (*p *= 0.021; OR = 2.30) are also associated with the risk of MI; the association of the biallelic combination with the risk of MI is less significant than MI association with the triallelic combination but more significant than MI association with the single LPL*G/G genotype (*Fig. 2B*). Another triallelic combination (APOE*ε4 + eNOS*T + rs1333049*C/G) is negatively associated with the risk of MI (*p *= 0.00041; OR = 0.30) (*Fig. 2C*). The association of the biallelic combinations being a part of it (APOE*ε4 + rs1333049*C/G) and (APOE*ε4 + eNOS*T) with the risk of MI is less significant. However, it is stronger than MI association with carriage of the APOE*ε4 allele, the only component of the combination that is significant alone. Hence, we have used multilocus analysis to identify that the genetic variants of the *MTHFR *gene (rs1801133) and 9p21 locus (rs1333049) within several allelic combinations are involved in predisposition to MI, while the genetic variants one by one showed no significant association with MI.

In order to answer the question what is the reason for the cumulative effect of the alleles of different genes (whether it is the summation of small mutually independent contributions of individual alleles or epistatic interactions between these alleles), we analyzed the three-way interactions using the statistical approach described earlier [6]. The synergy factor (SF) with 95% CI and the *p *values calculated using the exact three-way test, similar to the OR with 95% CI and the *p *value determined by standard evaluation of the associations between the phenotype and the genotype (i.e., using the two-way Fisher’s test), were not significant. Therefore, no significant epistatic interactions were revealed between the components of all the identified combinations.

**Table 2:** Distribution of alleles and genotypes of polymorphic regions of the examined genes in MI patients (n = 405) and controls (n = 198)

Carriage of alleles and genotypes	Patients, n (%)	Controls, n (%)	p	p_corr_^**^	OR (95% CI) for significant differences^**^
rs562556 in PCSK9					
A	389(96)	193(97)	NS	NS	
G	102(25)	65(33)	0.013	NS	0.69 (0.47–1.00)
A/A	303(75)	133(67)	0.013	NS	1.45 (1.00–2.10)
A/G	86(21)	60(30)	0.010	NS	0.62 (0.42–0.91)
G/G	16(4)	5(3)	NS	NS	
rs7412, rs429358 (epsilon polymorphism) in APOE					
ε2	63(16)	30(15)	NS	NS	
ε3	393(98)	194(98)	NS	NS	
ε4	40(10)	38(19)	0.0013	0.0091	0.46 (0.28–0.75)
ε2/ε2	5(1)	2(1)	NS	NS	
ε2/ε3	55(14)	26(13)	NS	NS	
ε2/ε4	3(1)	2(1)	NS	NS	
ε3/ε3	305(75)	132(67)	0.017	NS	1.52 (1.05–2.2)
ε3/ε4	33(8)	36(18)	0.00033	0.0023	0.40 (0.24–0.66)
ε4/ε4	4(1)	0(0)			
rs320 in LPL					
G	192(47)	85(43)	NS	NS	
T	363(90)	187(94)	0.032	NS	0.51 (0.25–0.99)
G/G	42(10)	11(6)	0.032	NS	0.51 (0.25–0.99)
G/T	150(37)	74(37)	NS	NS	
T/T	213(53)	113(57)	NS	NS	
rs1801133 in MTHFR					
C	369(91)	174(87)	NS	NS	
G	206(51)	106(54)	NS	NS	
C/C	199(49)	92(46)	NS	NS	
C/T	170(42)	82(41)	NS	NS	
T/T	36(9)	24(13)	NS	NS	
rs2070744 in eNOS					
C	253(62)	100(50)	0.0034	0.024	1.63 (1.16–2.30)
T	343(85)	174(88)	NS	NS	
C/C	62(15)	24(12)	NS	NS	
C/T	191(47)	76(38)	NS	NS	
T/T	152(38)	98(50)	0.0034	0.024	0.61 (0.43–0.86)
rs1333049 in the 9p21 region					
C	313(78)	155(78)	NS	NS	
G	305(75)	146(74)	NS	NS	
C/C	100(25)	52(26)	NS	NS	
C/G	213(53)	103(52)	NS	NS	
G/G	92(22)	43(22)	NS	NS	

NS – not significant.

^*^The Bonferroni correction for the number of tests (multiple comparisons) was applied to the p values.

^**^p < 0.05.

**Fig. 3:**
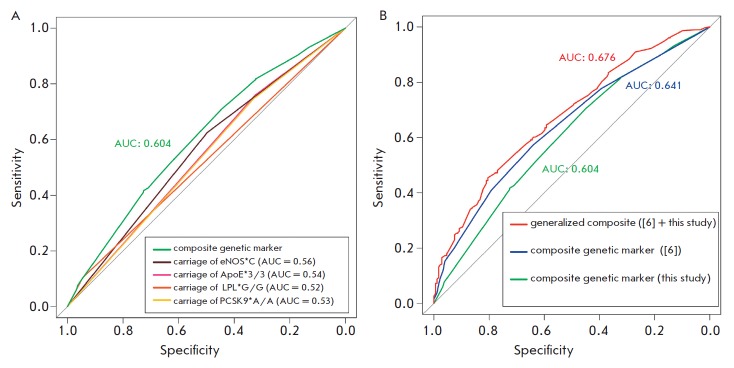
ROC analysis for efficiency of the models that are based on different genetic markers of individual risk of MI. A. The efficiency of classification of individuals using the models based on the carriage of individual genetic markers (variants of the *PCSK9*, *APOE*, *LPL*, and *eNOS *genes) and the model that takes into account the carriage of variants of all four genes (composite genetic marker, green line). B. Prognostic efficacy of the generalized composite model of individual genetic risk of MI (red line) obtained by supplementing the previously described model [6] (blue line) with data on the carriage of one or more variants of the *PCSK9*, *LPL*, *eNOS *and *APOE *genes (green line). The AUC (area under the curves) values for different models are shown in the same color as the corresponding curve.

In order to assess the prognostic significance of the identified genetic risk factors, we calculated the individual risk of MI in each subject depending on carriage of the *PCSK9*, *APOE, LPL, *and *eNOS *genetic variants using logistic regression. The contribution of carriage of a combination of these risk alleles/genotypes was evaluated using ROC analysis (*Fig. 3A*) according to the efficiency of classifying the subjects into MI patients and healthy individuals. One can see that the genetic factors considered one by one are poor classifiers of the risk of MI (AUC < 0.60). However, satisfactory prognostic efficacy is achieved (AUC = 0.604) when taking into account the data on carriage of a combination of the *PCSK9*, *APOE*, *LPL, *and *eNOS *alleles/genotypes. We would like to mention that the model does not become more efficient if the *MTHFR *and the 9p21 region alleles, which are the components of the combinations identified by APsampler, are added one by one.

These findings were used to improve the earlier built composite genetic model of the risk of MI, which includes the *TGFB1*, *FGB, *and *CRP *genetic variants and the epistatic combination of the *IFNG *and *PTGS1 *genes as predictors [6]. *Figure 3B* shows three ROC curves: the ROC curve obtained in the analyzed sample for the composite model described in [6]; the ROC curve for the carriage of a combination of the newly identified markers shown in *Fig. 3A*; and the ROC curve for the generalized composite model that includes the markers from both the previous and the present studies. One can see that the prognostic efficacy has significantly increased (*p *= 0.014): from AUC = 0.641 in the model without new markers to 0.676 in the generalized composite model.

## DISCUSSION

The case-control multilocus analysis of the association of the polymorphic variants of *PSCK9 *(rs562556), *APOE *(epsilon polymorphism, rs7412 and rs429358), *LPL *(rs320), *MTHFR *(rs1801133), *eNOS *(rs2070744), and the 9p21 region (rs1333049) with the risk of MI revealed the *PSCK9*, *APOE, LPL, *and *eNOS *alleles/genotypes significantly associated with MI and the bi- and triallelic combinations that carried the *MTHFR *and the 9p21 region alleles/genotypes along with the variants of the aforelisted genes.

Each of the examined lipid metabolism genes (*PCSK9*, *LPL*, and *APOE*) turns out to be associated with the risk of MI; the OR of the risk genotypes ranges from 1.45 to 1.96 but the significance level is rather low (*p *= 0.013–0.032). The involvement of the lipid metabo lism genes in the development of MI shows good agreement with the well-known fact that disorders of lipid metabolism, high cholesterol level and elevated atherogenic index lead to formation of atheromatous plaques in the arterial tunica intima. However, the published data regarding the involvement of the examined variants of the lipid metabolism genes in development of CVDs are rather controversial.

SNP rs562556 in the *PCSK9 *gene is responsible for the Ile-474-to-Val substitution in the encoded protein, which apparently does not affect its expression level [26]. We observed the MI association with the A/A genotype in this SNP in Russians. However, the distributions of rs562556 variants in Japanese subjects with MI were not different from those in the control population, although this SNP was associated with cholesterol level [27]. An association between polymorphism rs562556, the presence of anti-phospholipid antibodies, and development of thrombosis (the risk factor of MI) was revealed in subjects carrying these antibodies [28]. The association of other *PCSK9 *gene variants (namely, rs11206510 [29] and rs11591147 [30]) with MI was demonstrated in different populations. Hence, our findings regarding the association of the *PCSK9 *gene with MI are consistent with the data published earlier.

Individual allelic variants of the *APOE *gene epsilon polymorphism are associated with CVDs (and with MI in particular) in almost all populations. The meta-analyses demonstrate that the ε4 allele is associated with the risk of MI, while the ε2 allele has a protective effect [31, 32]. However, the conclusions drawn in the meta-analysis involving different ethnicities cannot be automatically extrapolated to separate populations, where the roles of individual alleles in predisposition to MI vary significantly. The considerable difference in allele frequencies in different populations and even within the same population residing in different regions is potentially the key reason for the poor replicability of the results obtained in individual studies [33]. In particular, ε3/ε3 turned out to be the risk genotype in our study involving Russians living in Central Russia, while ε2/ε3 was the risk genotype among Siberian males [34].

The HindIII polymorphism (rs320) is responsible for the T-to-G substitution in intron 8 of the *LPL *gene. This polymorphism is believed to reside in the regulatory sequence and to regulate *LPL *expression [35]. We revealed an association between rs320 and MI in our sample. The association between this polymorphism and the development of MI was demonstrated in a number of previous publications [36, 37], including the studies involving Russian populations [38]. Other polymorphisms of this gene associated with MI have been reported for the Japanese population [39]. However, the data on association of individual alleles with MI are sometimes inconsistent.

Polymorphism rs2070744 in the *eNOS *gene is another locus whose variant showed significant association with MI in our study. This polymorphism resides within the promoter region. The C allele associated with the risk of MI in our study is related to downregulation of mRNA expression and, correspondingly, the eNOS protein level [40]. Our data are consistent with the findings reported in other publications [41].

The C-to-T substitution in the rs1801133 variant of the *MTHFR *gene caused Ala-222-to-Val substitution in the protein [42] and reduction of methylenetetrahydrofolate reductase activity by almost 50% [43]. No association between rs1801133 and the risk of MI was found in different ethnic groups, including Caucasians [44] and Russians [45], in most studies. We also revealed no association of SNP rs1801133 with the risk of MI in Russians; however, carriage of the C allele in combination with the C allele of the *eNOS *gene (*Fig. 2A*) or carriage of the G/G genotype of the *LPL *gene (*Fig. 2B*) showed significant association with the risk of MI. We believe that these data can be interpreted as an argument in favor of the involvement of the *MTHFR* gene in predisposition to MI.

The 9p21 region is the only genomic region whose association with the risk of MI has been replicated in several GWASs at a genome-wide significance level (*p * < 5 × 10^-8^) [2]. These findings have been confirmed in a number of validation studies, including those for rs10757278 and rs1333049 in a sample consisting of MI patients and controls from the Siberian population (of unspecified ethnicity) [46]. In our study, no significant MI association with rs1333049 was found in Russians, but the multilocus analysis revealed a number of combinations containing this SNP. Carriage of the rs1333049*C allele within the triallelic combination is associated with the risk of MI (*Fig. 2B*), while the rs1333049*C/G genotype within the bi-and triallelic combinations was found to have a protective effect (*Fig. 2C*) and showed good agreement with the results reported in [46].

The statistical analysis of three-way interactions revealed no epistatic interactions between the components of all the identified combinations. Meanwhile, all the biallelic combinations associated with the risk of MI (OR > 1) are characterized by higher significance levels and higher OR values compared to those of alleles/ genotypes within these combinations considered one by one (correspondingly, lower OR values for the protective combinations with OR < 1). A similar regularity is observed for the triallelic combinations as compared to the biallelic ones. Therefore, the cumulative effects observed in this study result from the additivity of the contributions from individual genes. The reason for this additivity is that statistical significance in a relatively small sample for the association between a combination of unidirectional weak genetic factors and the disease is higher compared to the association observed for each factor one by one. Therefore, there is every reason to believe that the *MTHFR *(rs1801133) and 9p21 (rs1333049) loci are independent risk factors of MI having weak effects. Statistical powder was insufficient to reveal significant associations of these factors with MI in the examined sample, while multilocus analysis compensated for this drawback.

Identically, since the effects of the *MTHFR *(rs1801133) and 9p21 (rs1333049) loci are weak, adding them to the composite genetic model of the risk of MI did not increase the prognostic efficacy of the model. It is worth mentioning that the epistatic combinations are better risk classifiers than the additive combinations. Indeed, the epistatic combination of the *IFNG *and *PTGS1 *genes was found to be one of the MI risk factors, while both components were not associated with the disease one by one [6]. Meanwhile, identifying the genetic variants within additive combinations may indicate the possibility of identifying the association of these genetic variants one by one with the disease in larger samples.

Although being statistically significant, the prognostic efficacy of the composite genetic model of the risk of MI built using the findings obtained in our study is rather low. This is also true for the earlier obtained model [6]. The AUC value of 0.676 was achieved by combining the two models; at the cut-off equal to 0.74 it corresponds to sensitivity of 0.80 and specificity of 0.45. Overall, neither the results of GWASs nor the findings obtained using the candidate gene approach currently make it possible to effectively predict development of MI using genetic analysis.

## CONCLUSIONS

The analysis of the association between the polymorphic regions of six candidate genes and MI showed that they are significantly associated with MI, either one by one or within combinations. We have replicated the association of the polymorphic variants of the *PCSK9*, *APOE, LPL*, *MTHFR*, and *eNOS* genes and the 9p21 region with MI in independent samples of Russians living in Central Russia. Since the variants of the same genes (rs1801133 in the *MTHFR *gene or rs1333049 in the 9p21 region), which are not significant one by one, were components of several different combinations (with no epistatic interactions between their components revealed), it is fair to conclude that the cumulative effect of the genes within a combination identified using multilocus analysis results from summation of their small independent contributions.

Inclusion of the identified markers to the previously reported model of individual genetic risk [6] significantly increases its prognostic efficacy, although our findings need to be replicated for an independent sample of Russians. In order to further increase the predictive power of the composite model, it should to be improved by including other genetic predictors of risk and refining the regression coefficients for larger samples.

## Abbreviations

AUC: area under curve in ROC analysis
GWAS: genome-wide association study
NO: nitrogen oxide
ROC: receiver operating characteristic
SNP: single nucleotide polymorphism
CI: confidence interval
CAD: coronary artery disease
MI: myocardial infarction
OR: odds ratio
PCR: polymerase chain reaction
PCR-SSP: polymerase chain reaction using allele-specific primers
CVD: cardiovascular disease
m.a.: mean age
